# Generation of transgene-free canker-resistant *Citrus sinensis* cv. Hamlin in the T0 generation through Cas12a/CBE co-editing

**DOI:** 10.3389/fpls.2024.1385768

**Published:** 2024-03-26

**Authors:** Hongge Jia, Ahmad A. Omar, Jin Xu, Javier Dalmendray, Yuanchun Wang, Yu Feng, Wenting Wang, Zhuyuan Hu, Jude W. Grosser, Nian Wang

**Affiliations:** ^1^ Citrus Research and Education Center, Department of Microbiology and Cell Science, Institute of Food and Agricultural Sciences (IFAS), University of Florida, Lake Alfred, FL, United States; ^2^ Citrus Research and Education Center, Horticultural Sciences Department, Institute of Food and Agricultural Sciences (IFAS), University of Florida, Lake Alfred, FL, United States; ^3^ Biochemistry Department, Faculty of Agriculture, Zagazig University, Zagazig, Egypt

**Keywords:** transgene-free genome editing, CRISPR, Cas12a, Citrus, Xanthomonas, citrus canker

## Abstract

Citrus canker disease affects citrus production. This disease is caused by *Xanthomonas citri* subsp. citri (Xcc). Previous studies confirmed that during Xcc infection, PthA4, a transcriptional activator like effector (TALE), is translocated from the pathogen to host plant cells. PthA4 binds to the effector binding elements (EBEs) in the promoter region of canker susceptibility gene *LOB1* (EBE_PthA4_-LOBP) to activate its expression and subsequently cause canker symptoms. Previously, the Cas12a/CBE co-editing method was employed to disrupt EBE_PthA4_-LOBP of pummelo, which is highly homozygous. However, most commercial citrus cultivars are heterozygous hybrids and more difficult to generate homozygous/biallelic mutants. Here, we employed Cas12a/CBE co-editing method to edit EBE_PthA4_-LOBP of Hamlin (*Citrus sinensis*), a commercial heterozygous hybrid citrus cultivar grown worldwide. Binary vector GFP-p1380N-ttLbCas12a:LOBP1-mPBE:ALS2:ALS1 was constructed and shown to be functional via Xcc-facilitated agroinfiltration in Hamlin leaves. This construct allows the selection of transgene-free regenerants via GFP, edits *ALS* to generate chlorsulfuron-resistant regenerants as a selection marker for genome editing resulting from transient expression of the T-DNA via nCas9-mPBE:ALS2:ALS1, and edits gene(s) of interest (i.e., EBE_PthA4_-LOBP in this study) through ttLbCas12a, thus creating transgene-free citrus. Totally, 77 plantlets were produced. Among them, 8 plantlets were transgenic plants (#Ham_GFP_1 - #Ham_GFP_8), 4 plantlets were transgene-free (#Ham_NoGFP_1 - #Ham_NoGFP_4), and the rest were wild type. Among 4 transgene-free plantlets, three lines (#Ham_NoGFP_1, #Ham_NoGFP_2 and #Ham_NoGFP_3) contained biallelic mutations in EBE_pthA4_, and one line (#Ham_NoGFP_4) had homozygous mutations in EBE_pthA4_. We achieved 5.2% transgene-free homozygous/biallelic mutation efficiency for EBE_PthA4_–LOBP in *C. sinensis* cv. Hamlin, compared to 1.9% mutation efficiency for pummelo in a previous study. Importantly, the four transgene-free plantlets and 3 transgenic plantlets that survived were resistant against citrus canker. Taken together, Cas12a/CBE co-editing method has been successfully used to generate transgene-free canker‐resistant *C. sinensis* cv. Hamlin in the T0 generation via biallelic/homozygous editing of EBE_pthA4_ of the canker susceptibility gene *LOB1.*

## Introduction

Citrus is grown worldwide as one of the most popular fruits, which can be eaten fresh or consumed as juice. However, global citrus production faces many biotic and abiotic challenges, including citrus bacterial canker and Huanglongbing, droughts, flooding, and freezes (Hu et al., 2014; Cimen and Yesiloglu, 2016; Dala-Paula et al., 2019; Wang, 2019). New citrus cultivars are urgently needed to undertake these challenges. CRISPR/Cas mediated genome editing is deemed the most promising approach to breed new citrus cultivars, owing to its short time requirement, precise genetic improvement and predictable results (Chen et al., 2019; Gao, 2021; Cao et al., 2022; Huang et al., 2022a). To date, SpCas9/gRNA from *Streptococcus pyogenes*, SaCas9/gRNA from *Staphylococcus aureus*, LbCas12a/crRNA from *Lachnospiraceae bacterium* and base editor derived from SpCas9/gRNA have been successfully adapted to modify citrus genomes for gene function study and new citrus cultivar breeding (Jia and Wang, 2014a; Jia et al., 2016; Peng et al., 2017; Zhang et al., 2017; Jia et al., 2017a, Jia et al., 2017b; LeBlanc et al., 2018; Zhu et al., 2019; Jia et al., 2019a; Dutt et al., 2020; Huang et al., 2020; Jia and Wang, 2020; Huang and Wang, 2021; Jia et al., 2021; Alquezar et al., 2022; Jia et al., 2022; Mahmoud et al., 2022; Parajuli et al., 2022; Yang et al., 2022; Huang et al., 2022b, 2023; Su et al., 2023).

CRISPR genome editing has been used to breed disease-resistant varieties for many crops (Caserta et al., 2020; Bowen et al., 2022; Liu et al., 2022). For citrus, a lot of work has been done for generating canker-resistant citrus cultivars with most focused on editing *Citrus sinensis lateral organ boundary 1* (*CsLOB1*), the citrus canker susceptibility gene (Hu et al., 2014). Interestingly, editing the *DOWNY MILDEW RESISTANCE 6 (DMR6)* gene, which encodes 2-oxoglutarate Fe(II)-dependent dioxygenases, in citrus also results in improved resistance to Xcc (Parajuli et al., 2022). Citrus canker is one of the most economically important citrus diseases worldwide and is caused by *Xanthomonas citri* subsp. citri (Ference et al., 2018). Previous studies demonstrate that during Xcc infection, PthA4, a transcriptional activator like effector (TALE), is transported from Xcc cells to host plant cells. Once inside the cell nucleus, PthA4 binds to the effector binding elements (EBEs) in the promoter region of *CsLOB1* (CsLOBP) to activate its expression and expression of downstream genes, which consequently leads to canker symptom formation (Hu et al., 2014; Zhang et al., 2016; Duan et al., 2018; Zou et al., 2021; de Souza-Neto et al., 2023). Therefore, editing either *CsLOB1* coding region or the EBE recognized by PthA4 (EBE_pthA4_) has been adopted to create canker-resistant citrus. For instance, canker-resistant Duncan grapefruit was developed through editing *CsLOB1* coding region using SpCas9/gRNA (Jia et al., 2017b). Moreover, canker-resistant Duncan grapefruit, Hamlin, Pummelo and Wanjincheng orange were created by disrupting EBE_pthA4_ or the TATA box of *CsLOB1* using spCas9/gRNA, LbCas12a/crRNA and base editor (Peng et al., 2017; Jia and Wang, 2020; Jia et al., 2022; Huang et al., 2022b). However, it must be kept in mind that all of the aforementioned canker-resistant genome-edited citrus plants are transgenic, which have not been commercialized owing to regulations and public perception concerns (Jia et al., 2006; Gong et al., 2021; Turnbull et al., 2021).

Transgene-free genome editing, on the other hand, is promising to overcome such issues. Multiple strategies have been developed to produce transgene-free plants after target-gene editing with CRISPR/Cas (Kocsisova and Coneva, 2023). Some examples include delivering DNA-free gene editing reagents such as ribonucleoproteins (Woo et al., 2015; Malnoy et al., 2016; Subburaj et al., 2016; Svitashev et al., 2016; Liang et al., 2017; Andersson et al., 2018; Su et al., 2023), novel delivery vectors such as viruses (Mei et al., 2019; Ma et al., 2020; Liu et al., 2023), unconventional selection methods to bypass integration of transgenes (Veillet et al., 2019; Alquezar et al., 2022; Huang et al., 2022b, 2023; Wei et al., 2023), graft-mobile editing systems (Yang et al., 2023), and so on. Initially, Alquezar et al. and Huang et al. independently developed transgene-free citrus using CBE-mediated editing (Alquezar et al., 2022; Huang et al., 2022b). Recently, two studies have been reported to generate transgene-free genome-edited citrus resisting against canker in the T0 generation. Su et al. took advantage of Cas12a/crRNA ribonucleoprotein to disrupt the coding region of *LOB1* (Su et al., 2023), and another study employed Cas12/CBE co-editing method to edit EBE_PthA4_-LOBP of pummelo, which is highly homozygous and relatively easy to work with (Huang et al., 2023). Notably, the latter took advantage of green fluorescent protein for selecting transgene-free transformants, *Agrobacterium*-mediated transient expression of cytosine base editor (CBE) to edit *ALS* encoding acetolactate synthase to confer herbicide chlorsulfuron resistance as a selection marker, and Cas12a/CRISPR RNA for editing gene(s) of interest (Huang et al., 2023). Intriguingly, Cas12/CBE co-editing method has been successfully employed for transgene-free genome editing of multiple plant species, including potato, tomato, and tobacco in addition to citrus (Huang et al., 2023).

In this study, we successfully used Cas12/CBE co-editing strategy to modify EBE_PthA4_-LOBP of sweet orange cv. Hamlin, a heterozygous hybrid between pummelo (*C. maxima*) and mandarin (*C. reticulata*) and an important citrus cultivar grown worldwide. Using this co-editing strategy, we have generated multiple transgene-free biallelic/homozygous EBE_PthA4_-LOBP Hamlin mutants, which are canker resistant and demonstrated its usefulness in genetic improvements of heterozygous commercial citrus varieties.

## Materials and methods

### Plasmid construction

SpCas9p is one version of codon-optimized SpCas9 (Ma et al., 2015b), which was successfully employed to edit citrus genome (Jia et al., 2021). From 35S-SpCas9p:DunLOBP (Jia et al., 2021), codon-optimized SpCas9p was amplified using primer Cas9p-5-*Afl*II (5′-AGGTCTTAAGGACAAGAAGTACTCGATCGGCCTCGCCATCG GCACCAACAGCGTCGGCTGGGCGGTGATCAC-3′) and Cas9p-3-*Mlu*I (5′-AGTCACGCGT CTTCTTTTTCTTAGCCTGTCCGGCCTT-3′). After digestion with *Afl*II and *Mlu*I, part of SpCas9p was cloned into nCas9-PBE vector from Addgene (Addgene plasmid #98164) to form nCas9-mPBE vector. nCas9-PBE (plant base editor) was used to perform base editing in rice, wheat and maize (Zong et al., 2017). To construct GFP-p1380N-CmYLCV-nCas9-mPBE, the *Bam*HI- *Eco*RI-cut nCas9-mPBE fragment was ligated with *Bam*HI- *Eco*RI-cut GFP-p1380N-CmYLCV-nCas9-PBE (Huang et al., 2023).

From 35S-SpCas9p:DunLOBP (Jia et al., 2021), the AtU6-26 promoter was amplified using AtU6-26-5-*Xho*I (5′-AGGTCTCGAGTCGTTGAACAACGGAAACTCGACTTGCCTT-3′) and AtU6-26-3-phos (5′-phosphorylated-aatcactacttcgactctagctgt-3′), and the sgRNA-ALSBE-NosT fragment was PCR-amplified using sgRNA-ALSBE1-P1 (5′-phosphorylated-GcaggtcccTcggaggatgatGTTTTAGAGCTAGAAATAGCAAGT-3′) and NosT-3-*Spe*I (5′-aggtactagTCCGATCTAGTAACATAGATGACA-3′). Through three-way ligation, *Xho*I-cut AtU6-26 and *Spe*I-digested sgRNA-ALSBE1-NosT were inserted into *Xho*I-*Xba*I-treated pUC-NosT-MCS to construct pUC-NosT-AtU6-26-sgRNA-ALSBE1. pUC-NosT-MCS was constructed previously, which harbors the *Xho*I-*Asc*I-*Xba*I-*Pme*I multiple enzyme sites (Jia et al., 2019a). From pUC-NosT-AtU6-26-sgRNA-ALSBE (Huang et al., 2023), the AtU6-26-sgRNA-ALSBE2 fragment was amplified using AtU6-26-5-*Xho*I and NosT-3-*Bsa*I (5′-ATTCGGTCTCCCATGTATGAT AATCATCGCAAGACCGGC-3′), and the AtU6-26-sgRNA-ALSBE1 was PCR-amplified using AtU6-26-5-*Bsa*I (5′-TCGAGGTCTCCCATGTCGTTGAACAACGGAAACTCGACTTGCCTT-3′) and NosT-3-*Spe*I from pUC-NosT-AtU6-26-sgRNA-ALSBE1. Through three-way ligation, *Xho*I-*Bsa*I-cut *Xho*I-AtU6-26-sgRNA-ALSBE2-*Bsa*I and *Bsa*I-*Spe*I-digested *Bsa*I-AtU6-26-sgRNA-ALSBE1-*Spe*I were inserted into *Xho*I-*Xba*I-treated pUC-NosT-MCS to construct pUC-NosT-AtU6-26-sgRNA-ALSBE2-ALSBE1. Subsequently, the *Eco*RI-NosT-AtU6-26-sgRNA-ALSBE2-AtU6-26-sgRNA-ALSBE1-*Pme*I fragment from pUC-NosT-AtU6-26-sgRNA-ALSBE2-ALSBE1 were cloned into *Eco*RI-*Pme*I-cut GFP-p1380N-CmYLCV-nCas9-mPBE to generate GFP-p1380N-CmYLCV-nCas9-mPBE:ALS2:ALS1.

**Figure 1:**
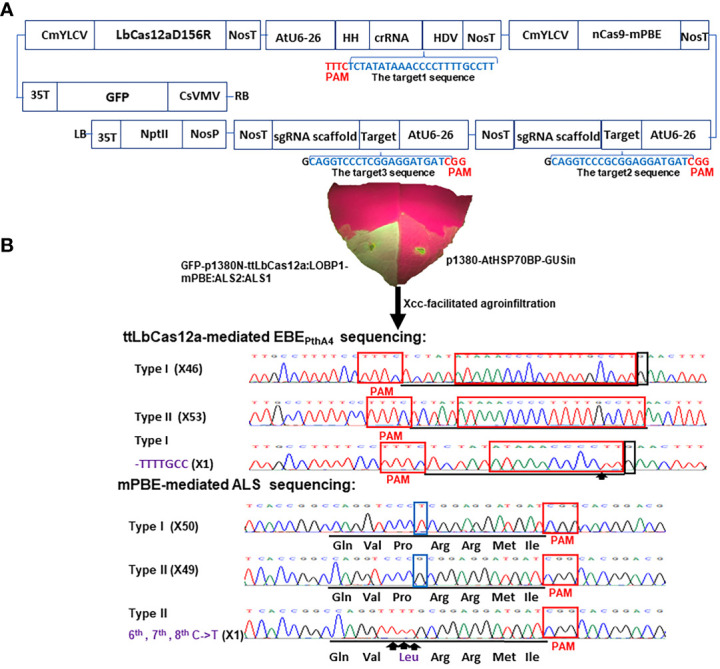
Schematic representation of the binary vector GFP-p1380N-ttLbCas12a:LOBP1-mPBE:ALS2:ALS1 and its functional test. **(A)** Schematic diagram of GFP-p1380N-ttLbCas12a:LOBP1-mPBE:ALS2:ALS1. *LOBP:* the promoter region of *LOB1.* LB and RB, the left and right borders of the T-DNA region; CsVMV, the cassava vein mosaic virus promoter; GFP, green fluorescent protein; 35T, the cauliflower mosaic virus 35S terminator; CmYLCV, the cestrum yellow leaf curling virus promoter; NosP and NosT, the nopaline synthase gene promoter and its terminator; ttLbCas12a, temperature-tolerant LbCas12a containing the single mutation D156R; AtU6-26, *Arabidopsis* U6-26 promoter; target1, the 23 nucleotides of Type II LOBP highlighted by blue, was located downstream of protospacer-adjacent motif (PAM); HH, the coding sequence of hammerhead ribozyme; HDV, the coding sequence of hepatitis delta virus ribozyme; nCas9-mPBE, a plant codon-optimized base editor composed of rat cytidine deaminase APOBEC1, Cas9-D10A nickase (nCas9) and uracil glycosylase inhibitor (UGI); AtU6-26, *Arabidopsis* U6-26 promoter; target2 and target3, the 20 nucleotides of two *CsALS* alleles highlighted by blue, were located upstream of protospacer-adjacent motif (PAM); NptII, the coding sequence of neomycin phosphotransferase II. **(B)** Xcc-facilitated agroinfiltration of the binary vector GFP-p1380N-ttLbCas12a:LOBP1-mPBE:ALS2:ALS1. Xcc-pre-treated Hamlin leaf was agroinfiltrated with *Agrobacterium* cells harboring vector GFP-p1380N-ttLbCas12a:LOBP1-mPBE:ALS2:ALS1. After four days, GFP fluorescence was observed and photographed. A negative control was *Agrobacterium* cells harboring p1380-AtHSP70BP-GUSin. ttLbCas12a-directed LOBP indels and mPBE-mediated *CsALS* base editing were analyzed through Sanger sequencing. Among 200 colonies sequenced, there were expected mutations for LOBP and *CsALS*. x inside the parentheses indicates number of Sanger sequencing. The targeted sequence within LOBP and *CsALS* was underlined by black lines, and the mutant site was pointed out with arrows and highlighted by purple. Type I LOBP has one more G than Type II LOBP downstream of EBE_PthA4_, and the G was highlighted by a black rectangle. The nucleotides different between two alleles of *CsALS* were highlighted by blue rectangles.

Finally, the *Asc*I-*Pme*I-cut CmYLCV-nCas9-mPBE:ALS2:ALS1 fragment GFP-p1380N-CmYLCV-nCas9-mPBE:ALS2:ALS1 was clone into *Asc*I-*Pme*I-cut vector GFP-p1380N-ttLbCas12a:LOBP1-*Asc*I-*Xba*I-*Pme*I, which was constructed previously (Huang et al., 2023), to form GFP-p1380N-ttLbCas12a:LOBP1-mPBE:ALS2:ALS1 (**Figure 1**, **Supplementary Figure 1**). Notably, GFP-p1380N-ttLbCas12a:LOBP1-mPBE:ALS2:ALS1 contained both GFP and *nptII* selectable genes that can be used for selection of putative non-transgenic transformants (**Figure 1**, **Supplementary Figure 1**) with GFP being an easier option for visual selection in our experience (Huang et al., 2023).

The binary vector GFP-p1380N-ttLbCas12a:LOBP1-mPBE:ALS2:ALS1 was transformed into *A. tumefaciens* strain EHA105 via electroporation. Recombinant *Agrobacterium* cells were employed for Xcc-facilitated agroinfiltration or citrus epicotyl transformation.

### *Xanthomonas citri* subsp. citri-facilitated agroinfiltration in Hamlin

Grown in a greenhouse at 28°C, *C. sinensis* cv. ‘Hamlin’ was pruned to generate uniform shooting before Xcc-facilitated agroinfiltration. It should be pointed out that ttLbCas12a performed better at 28°C than at 22°C in a previous study (Schindele and Puchta, 2020).

Xcc-facilitated agroinfiltration was performed as described previously (Jia and Wang, 2014b). Briefly, the fully-expanded young Hamlin leaves were first treated with Xcc, which was re-suspended in sterile tap water at a concentration of 5 × 10^8^ CFU/mL. Twenty-four hours later, the Xcc-treated leaf areas were inoculated with *Agrobacterium* cells harboring vectors GFP-p1380N-ttLbCas12a:LOBP1-mPBE:ALS2:ALS1, or p1380-AtHSP70BP-GUSin. p1380-AtHSP70BP-GUSin was constructed before (Jia and Wang, 2014b), and used as a no-GFP-fluorescence control. Four days after agroinfiltration, GFP was observed and photographed, and genomic DNA was extracted from the Hamlin leaves treated by agroinfiltration.

### *Agrobacterium*-mediated Hamlin transformation

As described previously (Jia et al., 2019b), Hamlin epicotyl transformation was conducted with minor modifications. Briefly, Hamlin epicotyl explants were co-incubated with *Agrobacterium* cells harboring the binary vector GFP-p1380N-ttLbCas12a:LOBP1-mPBE:ALS2:ALS1. After cocultivation in darkness for 3 days at 25°C, the epicotyl explants were placed on regeneration medium containing 50 mg/L kanamycin for one week at 28°C, and then the epicotyl explants were placed on regeneration medium containing 40 μg/L chlorsulfuron at 28°C (Veillet et al., 2019). All regenerated plants were subjected to genome editing analysis and GFP inspection. It is noteworthy that we initially placed Hamlin epicotyls on medium with 100 mg/L kanamycin for two weeks; three rounds of selection with 40 ng/mL chlorsulfuron, each lasting two weeks as reported previously (Veillet et al., 2019). However, very few shoots were regenerated. Based on our observations, we adapted the protocol by reducing the kanamycin concentration to 50 mg/L for one week, and conducting two rounds of selection with 40 ng/mL chlorsulfuron, each extending to three weeks.The GFP-positive and transgene-free regenerated shoots were selected and micro-grafted on ‘Carrizo’ citrange rootstock plants (*C. sinensis* (L.) Osbeck x *Poncirus trifoliata* (L.) Raf.) for further analysis. Six months later, they were used for PCR analysis with the primers Npt-Seq-5 (5′-TGTGCTCGACGTTGTCACTGAAGC-3′) and 35T-3 (5´-TTCGGGGGATCTGGATTTT AGTAC-3′).

### PCR amplification of mutagenized *CsALS* and LOBP

Genomic DNA was extracted from the Xcc-facilitated-agroinfiltrated Hamlin leaves or each regenerated Hamlin line. To analyze GFP-p1380N-ttLbCas12a:LOBP1-mPBE:ALS2:ALS1-mediate *CsALS* and LOBP mutations, PCR was carried out using either primers CsALSP1 (5′- Atctgtatcgccacctcggggcccggc-3′) and CsALSP2 (5′- TGGCCGCCCAGATGTTGCTAAAAGG-3′), or primers LOB21 (5′- ACACCTTGGTAATTTTGACATTAGGTA-3′) and LOB22 (5′- TGAGAGAAGAAAACTGTTGGGTTGTAG-3′). The PCR products were sequenced through cloning and colony sequencing. Primers CsALSP1 and CsALSP2 were designed to amplify the sgRNA-target *CsALS* region for the mutation analysis via Sanger sequencing, and primers LOB21 and LOB2 were employed to analyze LOBP. For PCR product direct sequencing, the CsALSP1/CsALSP2-amplified and LOB21/LOBP22-amplified PCR products were purified and subjected to sequencing using CsALSP3 (5′- tggtcagcgggctcgccgacgcgct-3′) as to *CsALS*, and primer LOB4 (5′-CGTCATTCAATTAAAATTAATGAC-3′) as to LOBP. Direct sequencing of PCR products was employed to genotype CRISPR/Cas-mediated indels (Ma et al., 2015a; Jia et al., 2016). Ten random colonies for each transgenic and transgene-free Hamin line were selected for sequencing. Mutation rates were calculated by the mutant colonies/10 colonies. Chromas Lite program was used to analyze the sequencing results.

### GFP detection

An Omax camera was installed onto a Zeiss Stemi SV11 dissecting microscope for photographing GFP fluorescence. Under illumination of the Stereo Microscope Fluorescence Adapter (NIGHTSEA), GFP fluorescence of the Hamlin leaves treated by Xcc-facilitated agroinfiltration and GFP-p1380N-ttLbCas12a:LOBP1-mPBE:ALS2:ALS1-transformed Hamlin was observed. Subsequently, the Hamlin leaves were photographed with the Omax Toupview software connected to the Omax camera.

### Canker symptom assay in citrus

Wild type, transgenic and transgene-free Hamlin plants were grown in a greenhouse at the Citrus Research and Education Center, University of Florida. Prior to Xcc inoculation, all plants were trimmed to generate new shoots. Leaves of similar age were infiltrated with either Xcc or XccΔpthA4:dLOB1.5 (5 × 10^8^ CFU/mL) using needleless syringes. At three, six and nine days post inoculation (DPI), canker symptoms were observed and photographed.

### Whole genome sequencing analysis of transgene-free Hamlin plant #Ham_NoGFP_4

Genomic DNA of transgene-free Hamlin plant #Ham_NoGFP_4 was subjected to whole genome sequencing at Novogene (Sacramento, CA, USA). The whole genome data of #Ham_NoGFP_4 were released at NCBI (NCBI Bio-project ID: PRJNA1073671). DNA Library construction, sequencing, and data analysis were performed as follows. Following the manufacturer’s protocol of short read DNA sequencing from Illumina (Kozarewa et al., 2009), the library was prepared. After quality control, quantification, and normalization of the DNA libraries, 150-bp paired-end reads were generated using the Illumina NovaSeq 6000 platform according to the manufacturer’s instructions at Novogene. The raw paired-end reads were filtered to remove the low-quality reads using fastp program version 0.22.0 (Chen et al., 2018). To assess the target site mutations of mutated plants, the high quality paired-end short genomic reads were mapped to sweet orange (*C. sinensis*) (Wang et al., 2021) reference genome using Bowtie2 software version 2.2.6 (Langmead and Salzberg, 2012). The mutations (single nucleotide polymorphisms, deletions and insertions) for the mutated plant genomes were identified using the SAMtools package version 1.2 (Li et al., 2009) and deepvariant program version 1.4.0 (Poplin et al., 2018). The identified mutations were filtered by quality and sequence depth (mapping quality > 10 and mapping depth > 10). The mutations of target sites were visualized using IGV software version 2.15.4 (Robinson et al., 2011). The off-target sites were predicted using CRISPR-P 2.0 (Liu et al., 2017) and the Cas-OFFinder (Bae et al., 2014) program and aligning target sequence with whole genome using blast program. Based on the mapping results, mutations of off-target sites were detected using the SAMtools package version 1.2 and deepvariant program version 1.4.0. To detect the potential foreign DNA contamination, the high quality paired-end short genomic reads were mapped to the plasmid sequences using Bowtie2 software version 2.2.6.

## Results

### Construction of the binary vector GFP-p1380N-ttLbCas12a:LOBP1-mPBE:ALS2:ALS1

In a previous study, GFP-p1380N-ttLbCas12a:LOBP1-PBE:ALS was constructed to produce transgene-free pummelo, which is a highly homozygous diploid (Wu et al., 2018). One sgRNA was designed to target both alleles of *acetolactate* synthase (*CsALS*) and another sgRNA for both alleles of EBE_PthA4_-LOBP in pummelo (Huang et al., 2023). *C. sinensis* cv. Hamlin is a heterozygous hybrid between pummelo (*C. maxima*) and mandarin (*C. reticulata*). In this study, the promoter region of *CsLOB1* (CsLOBP) from mandarin was designated as Type I LOBP, whereas CsLOBP from pummelo was named as Type II LOBP. As a result, Hamlin has Type I LOBP and Type II LOBP. Notably, Type I LOBP has one more G than Type II LOBP downstream of EBE_PthA4_, whose detailed sequence is ATAAACCCCTTTTGCCTT (**Figure 1**). Similarly, two alleles of *CsALS* from mandarin and from pummelo were named as Type I *CsALS* and Type II *CsALS*, respectively. As a result, Hamlin also has Type I *CsALS* and Type II *CsALS*. Notably, Type I *CsALS* has T at the position of 9, and Type II *CsALS* has G (the PAM labeled as positions from 21 to 23) (**Figure 1**). Consequently, two different sgRNAs were designed to target the *ALS* gene of Hamlin in vector GFP-p1380N-ttLbCas12a:LOBP1-mPBE:ALS2:ALS1 (**Figure 1**). On the other hand, one crRNA was designed to target the conserved sequence of both alleles of EBE_PthA4_-LOBP of Hamlin. Based on the reference genome of sweet orange genome (V3) from HZAU (http://citrus.hzau.edu.cn/index.php) (Liu et al., 2017), the genomic position for target 1 (TCTATATAAACCCCTTTTGCCTT) is from 3970724-3970746 on chromosome 7. The genomic positions for target 2 (caggtcccGcggaggatgat) and targets 3 (caggtcccTcggaggatgat) are from 15794167-15794186 on chromosome 7, since target 2 and targets 3 are two alleles of *CsALS* (**Figure 1A**). To enhance editing efficiency, base editor nCas9-PBE of GFP-p1380N-ttLbCas12a:LOBP1-PBE:ALS was optimized to form nCas9-mPBE in GFP-p1380N-ttLbCas12a:LOBP1-mPBE:ALS2:ALS1, which harbors the codon-optimized SpCas9p backbone for plants (**Figure 1A**). SpCas9p has been used to produce homozygous/biallelic mutations for multiple plant species in the T0 generation, such as Arabidopsis, rice, and citrus (Ma et al., 2015b; Jia and Wang, 2020).

### Evaluation of GFP-p1380N-ttLbCas12a:LOBP1-mPBE:ALS2:ALS1 efficacy via Xcc-facilitated agroinfiltration

Xcc-facilitated agroinfiltration of Hamlin leaf was used to test whether GFP-p1380N-ttLbCas12a:LOBP1-mPBE:ALS2:ALS1 could be employed to edit citrus EBE_PthA4_-LOBP by ttLbCas12a and edit *CsALS* by nCas9-mPBE. It must be pointed out that cestrum yellow leaf curling virus (CmYLCV) promoter was used to drive ttLbCas12a and nCas9-mPBE expression in GFP-p1380N-ttLbCas12a:LOBP1-mPBE:ALS2:ALS1 (**Figure 1**, **Supplementary Figure 1**), since CmYLCV outperformed CaMV 35S for citrus genome editing (Huang et al., 2022a). In addition, in order to promote editing, the coding sequence of hammerhead ribozyme (HH) at the 5’ end of crRNA, and the coding sequence of hepatitis delta virus ribozyme (HDV) were placed at the 3’ end of crRNA (**Figure 1**, **Supplementary Figure 1**) (Tang et al., 2017). Genomic DNA was extracted from GFP-expressing Hamlin leaf and subjected to PCR, ligation and *E. coli* transformation. Sanger sequencing results showed that one colony contained ttLbCas12a-directed indels in EBE_PthA4_-LOBP among one batch of 100 colonies sequenced, and one colony contained the nCas9-PBE-mediated cytosine-to-thymine base conversion in *CsALS* among another batch of 100 colonies sequenced (**Figure 1B**). Therefore, GFP-p1380N-ttLbCas12a:LOBP1-mPBE:ALS2:ALS1 was functional to edit both EBE_PthA4_-LOBP and *CsALS* in Hamlin. Though the same genomic DNA was used as PCR template, two pairs of primers, CsALSP1/CsALSP2 and LOB21/LOBP22, were separately used to analyze mutant *CsALS* and LOBP. It is likely that ttLbCas12a-directed indels in LOBP and the nCas9-PBE-mediated cytosine-to-thymine base conversion in *CsALS* took place in the same cells even though we could not totally exclude other possibilities.

### Generation of transgene-free EBE_PthA4_-LOBP edited Hamlin via the co-editing method

Hamlin epicotyls were transformed with recombinant *Agrobacterium* cells harboring GFP-p1380N-ttLbCas12a:LOBP1-mPBE:ALS2:ALS1 (**Figure 1**) (Jia et al., 2019b). Three-day after co-cultivation, the epicotyls were first selected on 50 mg/L kanamycin for one week, then transferred to selective medium containing 40 μg/L chlorsulfuron for six weeks, during which new chlorsulfuron-containing medium was used after three-week cultivation. After six week herbicide selection, Hamlin epicotyls were cultivated on chlorsulfuron-free medium. In addition, the shoot generation was done at 28°C to facilitate LbCas12aD156-mediated EBE_PthA4_-LOBP editing (Schindele and Puchta, 2020; Jia et al., 2022).

**Figure 2:**
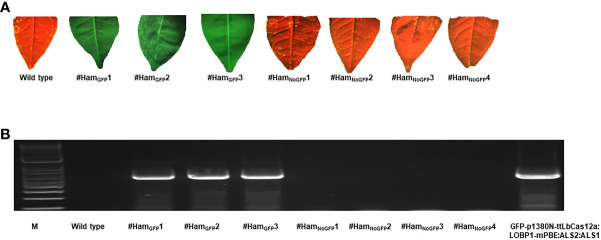
GFP detection and PCR verification of GFP-p1380N-ttLbCas12a:LOBP1-mPBE:ALS2:ALS1-transformed and transgene-free genome-edited Hamlin plants. **(A)** GFP fluorescence was observed in transgenic Hamlin plants, whereas wild type and transgene-free genome-edited plants did not show GFP. **(B)** Using a pair of primers Npt-Seq-5 and 35T-3PCR, wild type, transgenic and transgene-free Hamlin plants were analyzed. The wild type Hamlin and plasmid GFP-p1380N-ttLbCas12a:LOBP1-mPBE:ALS2:ALS1 were used as controls. M, 1kb DNA ladder.

In the presence of chlorsulfuron, 77 shoots were established. Among them, 8 shoots were GFP-positive, named as #Ham_GFP_1 to #Ham_GFP_8. Unexpectedly, five lines (#Ham_GFP_4, #Ham_GFP_5, #Ham_GFP_6, #Ham_GFP_7 and #Ham_GFP_8) died after grafting, however, three GFP-positive shoots (#Ham_GFP_1, #Ham_GFP_2 and #Ham_GFP_3) survived (**Figure 2A**). On the other hand, 69 shoots had no GFP expression, designated as #Ham_NoGFP_1 to #Ham_NoGFP_69 (**Figure 2A**).

**Figure 3:**
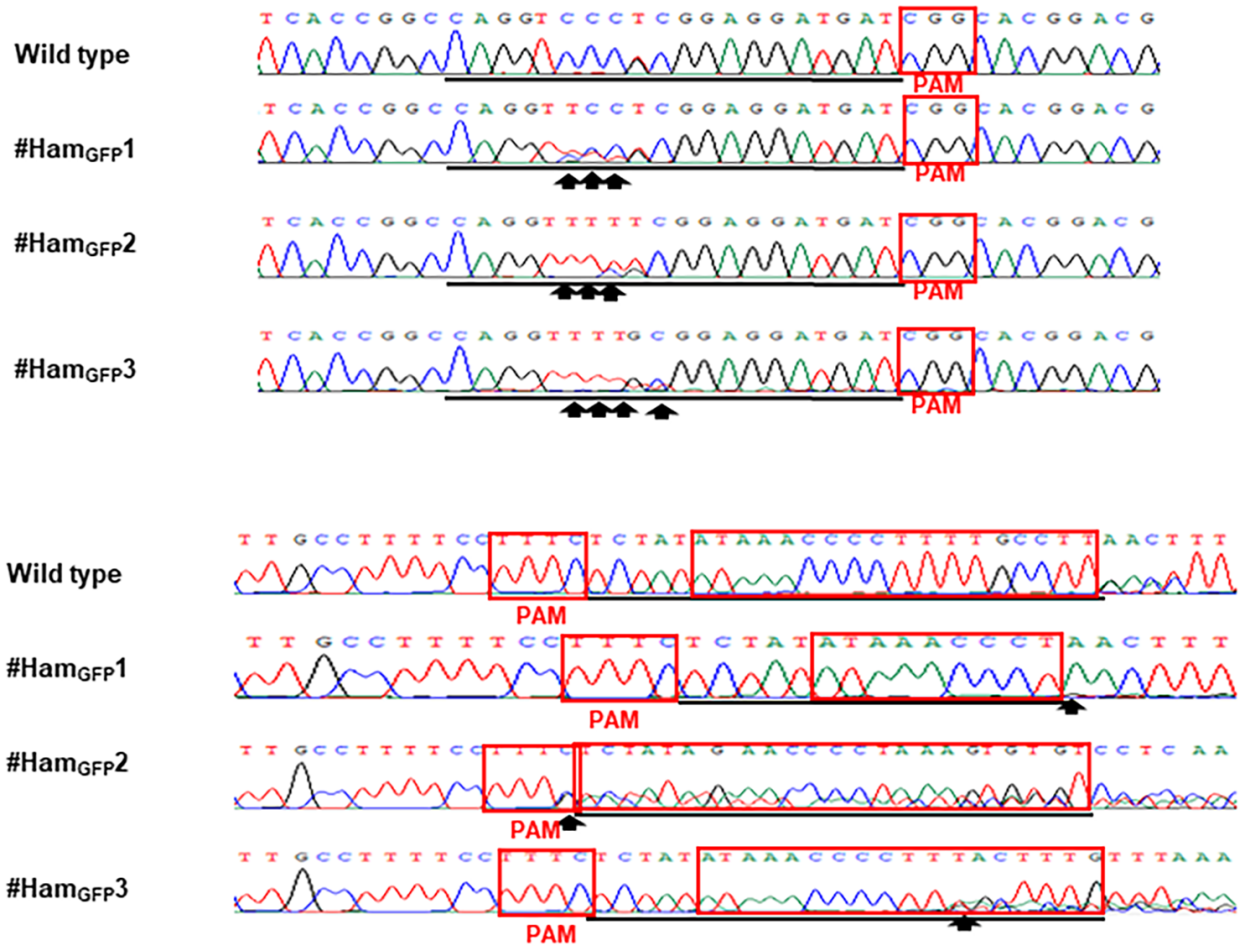
Detection of genome editing of GFP-p1380N-ttLbCas12a:LOBP1-mPBE:ALS2:ALS1-transformed Hamlin by direct sequencing of *CsALS* PCR products **(A)** and LOBP PCR products **(B)**. **(A)** The chromatograms of direct sequencing of *CsALS* PCR products. Primers CsALSP1 and CsALSP2 were used to amplify *CsALS* from wild type and transgenic Hamlin. Direct sequencing primer was CsALSP3. The base editing sites were shown by arrows. **(B)** The chromatograms of direct sequencing of LOBP PCR products. Primers LOB21 and LOB22 were used to amplify LOBP from wild type and transgenic Hamlin. Direct sequencing primer was LOB4. The mutation site or the beginning sites of multiple peaks were shown by arrows. The targeted sequence was underlined by black lines and EBE_PthA4_-LOBP was highlighted by red rectangles.

**Figure 4:**
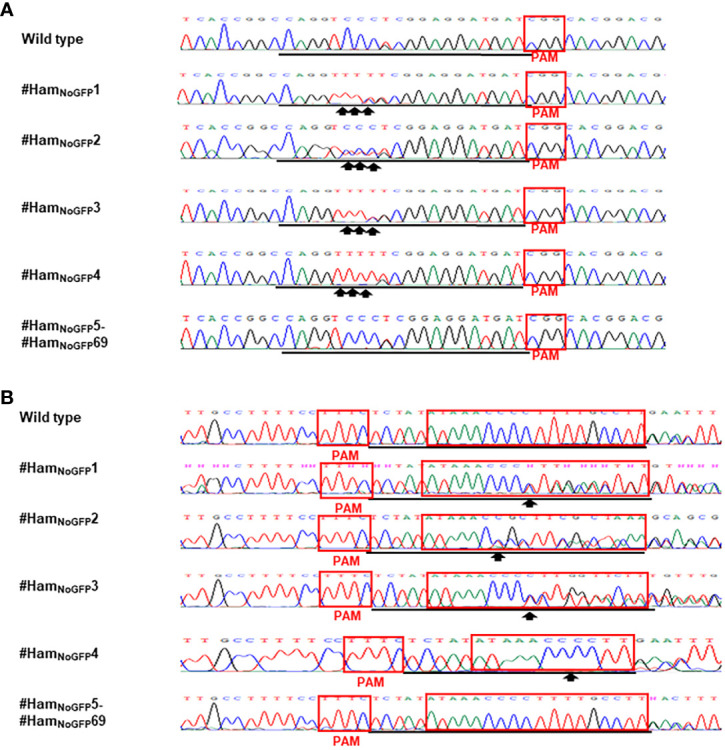
Detection of genome editing of no-GFP-expressing Hamlin by direct sequencing of *CsALS* PCR products **(A)** and LOBP PCR products **(B)**. **(A)** The chromatograms of direct sequencing of CsALS PCR products. Primers CsALSP1 and CsALSP2 were used to amplify *CsALS* from wild type and transgenic Hamlin. Direct sequencing primer was CsALSP3. The base editing sites were shown by arrows. **(B)** The chromatograms of direct sequencing of LOBP PCR products. Primers LOB21 and LOB22 were used to amplify LOBP from wild type and transgenic Hamlin. Direct sequencing primer was LOB4. The mutation site or the beginning sites of double peaks were shown by arrows. The targeted sequence was underlined by black lines and EBE_PthA4_-LOBP was highlighted by red rectangles.

Based on the results of direct sequencing of PCR products from transgenic Hamlin, ttLbCas12a-directed indels and mPBE-mediated C-to-T conversions were observed in #Ham_GFP_1, #Ham_GFP_2 and #Ham_GFP_3 (**Figure 3**). Among the 69 no-GFP-expressing shoots, four shoots, #Ham_NoGFP_1 to #Ham_NoGFP_4, had mutations in *CsALS* and EBE_PthA4_-LOBP (**Figure 4**), whereas the rest, from #Ham_NoGFP_5 to #Ham_NoGFP_69, had wild type *CsALS* and EBE_PthA4_-LOBP (**Figure 4**). Therefore, the four no-GFP-expressing lines (#Ham_NoGFP_1, #Ham_NoGFP_2, #Ham_NoGFP_3, #Ham_NoGFP_4) are likely transgene-free EBE_PthA4_-LOBP-edited lines.

To further verify whether the four no-GFP-expressing lines are transgene-free, we analyzed *nptII* gene in GFP-positive shoots (#Ham_GFP_1 to #Ham_GFP_3) and no-GFP-expressing shoots (#Ham_NoGFP_1 to #Ham_NoGFP_4). Using a pair of primers Npt-Seq-5 and 35T-3, *nptII* gene was subjected to PCR analysis. Three GFP-positive Hamlin had the expected *nptII* PCR products (**Figure 2B**), which verified that #Ham_GFP_1 to #Ham_GFP_3 were transgenic. No *nptII* PCR products were observed in no-GFP-expressing Hamlin lines from #Ham_NoGFP_1 to #Ham_NoGFP_4. The results confirmed that #Ham_NoGFP_1, #Ham_NoGFP_2, #Ham_NoGFP_3, and #Ham_NoGFP_4 were transgene-free plants (**Figure 2B**).

Sanger sequencing was employed to analyze mutation genotypes of the edited lines through cloning. As for EBE_PthA4_-LOBP, the results demonstrated that #Ham_GFP_1, #Ham_GFP_2 and #Ham_GFP_3 contained biallelic, chimeric and chimeric mutations, respectively (**Supplementary Figures 2**, **3**). As for *ALS*, #Ham_GFP_1, #Ham_GFP_2 and #Ham_GFP_3 contained chimeric, biallelic and biallelic mutations, respectively (**Supplementary Figures 2**, **3**). Intriguingly, #Ham_GFP_3 harbored C to T substitutions at C_10_ of *CsALS* sgRNA (**Supplementary Figure 3A**), which is somehow out of nCas9-PBE-mediated C-to-T conversion ranges from position 3 to 9 within the protospacer, counting the PAM as positions 21-23 (Zong et al., 2017). Previous study already showed that CBE could convert C to T outside the editing window (Lv et al., 2020). In addition, 7 colonies had the C to T substitution at C10 among 10 colonies sequenced. Thus, the C to T substitutions at C_10_ of *CsALS* in #Ham_GFP_3 could attribute to nCas9-PBE activity rather than sequencing errors. #Ham_GFP_2 contained 24 bps deletions from EBE_PthA4_-LOBP, including part of PAM (**Supplementary Figure 2D**).

**Figure 5:**
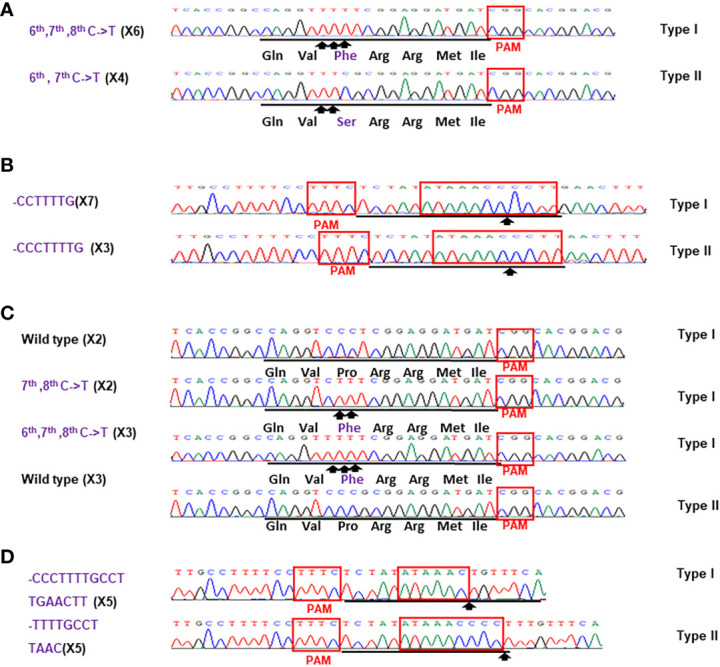
Sanger analysis of #Ham_NoGFP_1 **(A, B)** and #Ham_NoGFP_2 (c, d). Sanger sequencing results of #Ham_NoGFP_1 and #Ham_NoGFP_2. **(A)** As for *CsALS* of #Ham_NoGFP_1, Type I allele contained 6^th^, 7^th^, 8^th^ C->T changes, and Type II allele was 6^th^, 7^th^ C->T mutant among 10 colonies sequenced. **(B)** As for EBE_PthA4_-LOBP of #Ham_NoGFP_2, Type I allele had CCTTTTG deletion, and Type II allele had CCCTTTTG deletion. **(C)** As for *CsALS* of #Ham_NoGFP_2, wild type and mutants were present among 10 colonies sequenced. **(D)** As for EBE_PthA4_-LOBP of #Ham_NoGFP_2, five of them are CCCTTTTGCCTTGAACTT deletion from Type I allele, and five of them are TTTTGCCTTAAC deletion from Type II allele.

**Figure 6:**
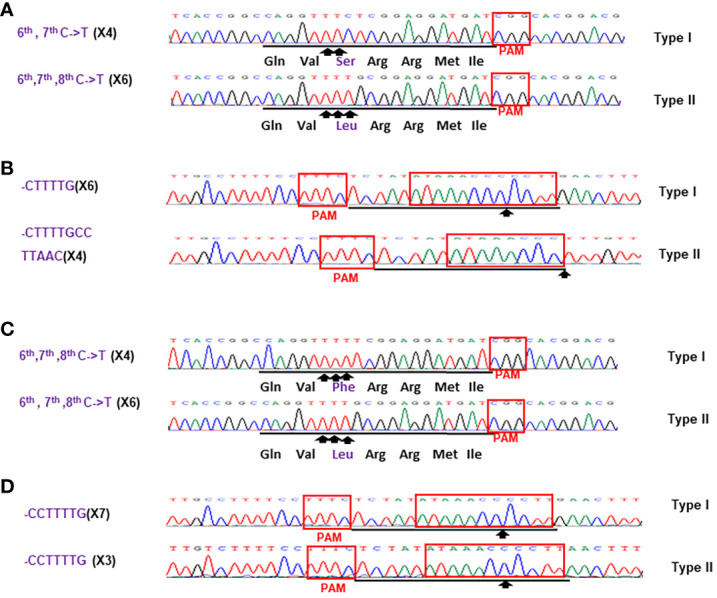
Sanger analysis of #Ham_NoGFP_3 **(A, B)** and #Ham_NoGFP_4 **(C, D)**. Sanger sequencing results of #Ham_NoGFP_3 and #Ham_NoGFP_4. **(A)** As for *CsALS* of #Ham_NoGFP_3, Type I allele was 6^th^, 7^th^ C->T mutant, and Type II allele had 6^th^, 7^th^, 8^th^ C->T mutation among 10 colonies sequenced. **(B)** As for EBE_PthA4_-LOBP of #Ham_NoGFP_3, Type I allele harbored CTTTTG deletion, and Type II allele contained CTTTtGCcttAAC deletion. **(C)** As for *CsALS* of #Ham_NoGFP_4, Type I and Type II allele were 6^th^, 7^th^, 8^th^ C->T mutant among 10 colonies sequenced. **(D)** As for EBE_PthA4_-LOBP of #Ham_NoGFP_4, Type I and Type II allele had CCTTTTG deletion.

For the transgene-free edited lines, Sanger sequencing results showed that #Ham_NoGFP_1, #Ham_NoGFP_2, #Ham_NoGFP_3 and #Ham_NoGFP_4 contained biallelic, biallelic, biallelic and homozygous mutations, respectively, in EBE_PthA4_-LOBP (**Figures 5B, D**, **6, B**). As for *ALS*, Sanger sequencing results indicated that #Ham_NoGFP_1, #Ham_NoGFP_2, #Ham_NoGFP_3 and #Ham_NoGFP_4 harbored biallelic, chimeric, biallelic and homozygous mutations, respectively (**Figures 5A, C**, **6A, C**). Notably, #Ham_NoGFP_4 had homozygous mutations in both EBE_PthA4_-LOBP and *CsALS* (**Figures 6C, D**).

Sanger sequencing results of transgenic and transgene-free Hamlin demonstrated that EBE_PthA4_-LOBP mutations were independent of those of *ALS*, which is consistent with the previous study in pummelo (Huang et al., 2023). ttLbCas12a deleted several bps from the target site, which occurred ≥10th bp distal to the PAM site except that of #Ham_GFP_2 (**Figure 5**, **6**, **Supplementary Figures 2**, **3**), which is consistent with previous work in citrus (Jia et al., 2022; Huang et al., 2023). The #Ham_GFP_2 harbored 24 bps deletions overlapping with PAM in EBE_PthA4_-LOBP (**Supplementary Figure 2D**). nCas9-mPBE catalyzed the targeted conversion of cytosine to thymine from position 6 to 8 within the protospacer except that of #Ham_GFP_3, which is consistent with previous work in rice, wheat and maize (Zong et al., 2017). Notably, the EBE_PthA4_-LOBP mutation rates were 100% in #Ham_GFP_1, #Ham_GFP_2, #Ham_NoGFP_1, #Ham_NoGFP_2, #Ham_NoGFP_3 and #Ham_NoGFP_4 (**Supplementary Figure 2**, **Figures 5**, **6**).

### Canker resistance of transgenic and transgene-free Hamlin plants

Since Hamlin plants from #Ham_NoGFP_5 to #Ham_NoGFP_69 had no mutations in EBE_PthA4_-LOBP (**Figure 4**), they were not tested with Xcc inoculation. Three transgenic plants (#Ham_GFP_1-3) and four transgene-free plants (#Ham_NoGFP_1-4) were evaluated for canker resistance. Wild type Hamlin was used as a control. All plants were inoculated with Xcc and XccΔpthA4:dLOB1.5 at a concentration of 5 × 10^8^ CFU/mL. XccΔpthA4:dLOB1.5 was included as a positive control because dLOB1.5 is a designed TALE, which binds to the sequence 5´- TAAAGCAGCTCCTCCTCATCCCTT- 3′ in the promoter region of *LOB1*, away from the EBE region, to activate *LOB1* expression (**Supplementary Figure 4A**) (Jia and Wang, 2020). Therefore, dLOB1.5 can mimic PthA4 function, and XccΔpthA4:dLOB1.5 can cause citrus canker, giving that its recognizing sequence is intact (Jia and Wang, 2020) whereasXccΔpthA4 cannot cause canker (Hu et al., 2014). Sanger sequencing results indicated that the dLOB1.5 binding sites were intact among all Hamlin plants (**Supplementary Figure 4B**).

**Figure 7:**
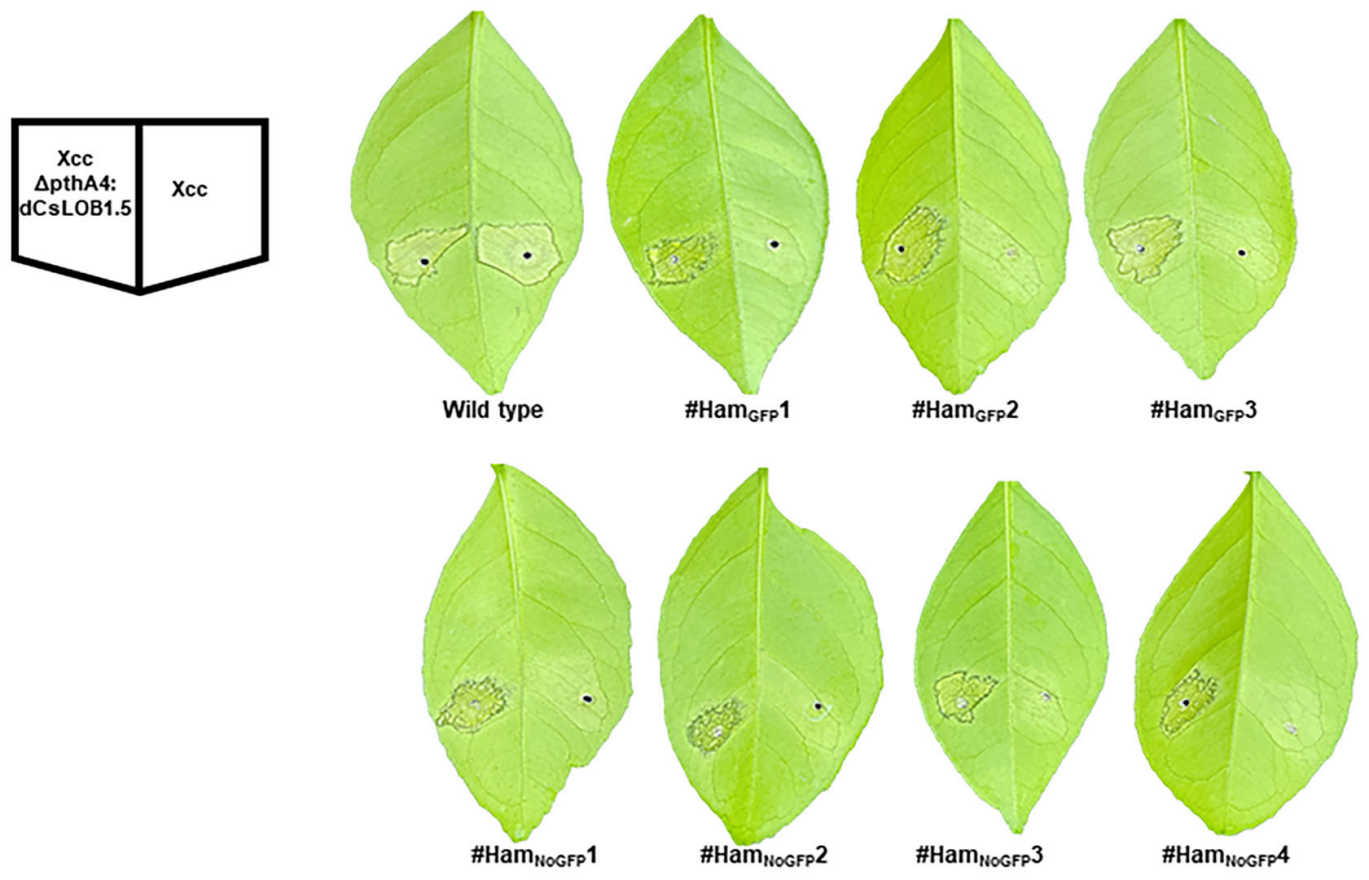
Canker-resistance in the transgenic and transgene-free EBE_PthA4_-LOBP-edited Hamlin plants. Six days post Xcc inoculation, citrus canker symptoms were observed on wild type Hamlin, whereas no canker symptoms were observed on LOBP-edited Hamlin plants. As expected, *XccpthA4:Tn5* (dCsLOB1.5) caused canker symptoms on all plants. dCsLOB1.5 induces *LOB1* to cause canker symptoms by recognizing a different region from EBE_PthA4_-LOBP.

At three, six and nine days post inoculation (DPI) with Xcc, canker symptoms were observed on wild type Hamlin plants, whereas no canker symptoms were observed on transgene-free #Ham_NoGFP_1, #Ham_NoGFP_2; #Ham_NoGFP_3, #Ham_NoGFP_4 and three transgenic Hamlin plants (**Figure 7**, **Supplementary Figure 5**). The results indicated that all plants, transgenic or not, harboring 100% mutations in EBE_PthA4_-LOBP (**Figures 5**, **6**, **Supplementary Figure 2**), resisted against Xcc infection as expected. Intriguingly, #Ham_GFP_3 containing 90% mutation in EBE_PthA4_-LOBP also resisted against Xcc (**Figure 7**, **Supplementary Figure 3**). Consistently, Cas9/sgRNA-transformed D_LOB_9 harboring 89.36% mutation rate in *CsLOB1* also showed Xcc resistance in a previous study (Jia et al., 2017a). At three, six and nine DPI with XccΔpthA4:dLOB1.5, canker symptoms developed on all treated plants (**Figure 7**, **Supplementary Figure 5**), since there was no editing in the dLOB1.5 binding sites (**Supplementary Figure 4B**). Therefore, it was the EBE_PthA4_-LOBP disruption that conferred Hamlin resistance against Xcc.

### Whole genome sequencing analysis of #Ham_NoGFP_4

To further confirm whether the transgene-free genome-edited lines are indeed free of plasmid sequences, we conducted a whole genome sequencing analysis of #Ham_NoGFP_4. #Ham_NoGFP_4 was selected because it contains homozygous EBE_PthA4_-LOBP and *CsALS* mutations (**Figures 6C, D**). We obtained high quality paired-end short genomic reads for #Ham_NoGFP_4 which were mapped to the T-DNA sequence corresponding to GFP-p1380N-ttLbCas12a:LOBP1-mPBE:ALS2:ALS1 using Bowtie2 software version 2.2.6. No T-DNA sequences were mapped to the genomic DNA of #Ham_NoGFP_4, confirming it was transgene-free. Furthermore, whole genome sequencing analysis indicated that #Ham_NoGFP_4 harbored homozygous mutations in both EBE_PthA4_-LOBP and *ALS* (**Supplementary Figure 6**), which was consistent with Sanger sequencing results (**Figure 6**).

To analyze off-target mutations, CRISPR-P 2.0 (Liu et al., 2017) and the Cas-OFFinder (Bae et al., 2014) program were used to find the potential off-targets of crRNAs and sgRNAs of GFP-p1380N-ttLbCas12a:LOBP1-mPBE:ALS2:ALS1. No potential off-target was identified for EBE_PthA4_-LOBP and *CsALS* (mismatch number ≤3). Since no potential off-target was identified for EBEPthA4-LOBP and *CsALS* (mismatch number ≤3), we changed the mismatch number ≤4. Consequently, 1 potential off-target was identified for EBEPthA4-LOBP, whereas 1 and 4 potential off-targets were identified for type I and type II *CsALS* alleles, respectively. However, analyses of the whole genome sequencing for the potential off-targets (mismatch number ≤4) did not identify any off-target mutations for both EBEPthA4-LOBP and *CsALS*.

## Discussion

In this study, we successfully employed the co-editing strategy, which couples ttLbCas12a with base editor nCas9-mPBE and GFP selection, to produce transgene-free canker-resistant Hamlin in the T0 generation via transient expression. For this purpose, the binary vector GFP-p1380N-ttLbCas12a:LOBP1-mPBE:ALS2:ALS1 was constructed (**Figure 1**, **Supplementary Figure 1**). This plasmid contains GFP, which facilitates the selection of transgene-free regenerants, nCas9-mPBE:ALS2:ALS1 which edits *ALS* to generate chlorsulfuron-resistant regenerants as a selection marker for genome editing resulting from transient expression of the T-DNA, and ttLbCas12a which edits gene(s) of interest (i.e., EBE_PthA4_-LOBP in this study). In addition, this plasmid contains *nptII* gene, which can also be used for selection of non-transgenic transformants. In a previous study, the co-editing method was used to generate transgene-free tobacco, tomato, potato, and pummelo (Huang et al., 2023). This study further demonstrates that the co-editing strategy can be used for genetic improvements of elite citrus varieties that are heterozygous hybrids via transgene-free genome editing in the T0 generation. In the previous study, two transgene-free homozygous/biallelic EBE_PthA4_–LOBP pummelo mutants were identified from 107 generated shoots, representing 1.9% transgene-free homozygous/biallelic mutation efficiency. Here, four transgene-free homozygous/biallelic EBE_PthA4_–LOBP *C. sinensis* cv. Hamlin mutants were identified from 77 generated shoots, representing 5.2% transgene-free homozygous/biallelic mutation efficiency. The improvement in transgene-free homozygous/biallelic mutation efficiency might result from the optimization of the base editor. The Cas9 nickase (nCas9) in the base editor construct of the previous study (Huang et al., 2023) was codon optimized for cereal crops which are monocots (Zong et al., 2017). Here, we have replaced the nCas9 with SpCas9p backbone which was codon optimized for both monocots and dicots, including citrus (Ma et al., 2015b; Jia and Wang, 2020).

To date, two methods have been employed to develop transgene-free citrus in the T0 generation: 1) PEG-mediated embryogenic protoplast infection with LbCas12aU/crRNA RNP (Su et al., 2023); 2) *Agrobacterium*-mediated epicotyl transformation with Cas12a/CBE co-editing (Huang et al., 2023). Both methods have their pros and cons. As for LbCas12aU/crRNA RNP method, all regenerants containing the target editing should be regarded as transgene-free, owing to no foreign DNA involved during PEG infection. Most importantly, RNP method had a very high biallelic/homozygous mutation rate, which is up to 97.4%. However, the reagents for LbCas12aU/crRNA RNP method were expensive, and 10 months were needed to establish canker-resistant Hamlin (Su et al., 2023). As for Cas12a/CBE co-editing method, the reagents related to *Agrobacterium* transformation were cheap, and 6 months were needed to establish canker-resistant citrus in this study. Since binary vector was used for epicotyl transformation, whole genome sequencing must be carried out to exclude potential T-DNA insertion in the chromosome (Huang et al., 2023). In addition, homozygous/biallelic mutation efficiency, which is 5.2% in this study, is much lower than that of RNP method. It is worthy to test whether PEG-mediated protoplast infection with Cas12a/CBE can produce transgene-free citrus with higher homozygous/biallelic efficiency.

In this study, one transgene-free line was chimeric in the *ALS* gene and 3 transgenic lines were chimeric in either the *CsALS* gene or the EBE_PthA4_–LOBP among the 77 regenerated shoots. Epicotyls were used as explants for Cas12a/CBE co-editing here, previous study indicated that a high frequency of chimeric shoots were observed when citrus epicotyl was the target explants for *Agrobacterium*-mediated transformation (Domínguez et al., 2004). Consistently, the chimeric/mosaic shoots were commonly developed during *Agrobacterium*-mediated citrus epicotyl transformation with CRISPR/Cas (Peng et al., 2017; Zhang et al., 2017). Remarkably, there were no regenerants containing chimeric *CsLOB1* when LbCas12aU/crRNA RNP was employed to infect protoplasts to develop transgene-free Hamlin (Su et al., 2023). The underneath mechanisms for the chimeric mutations in the two methods remain to be explored.

Transgene-free genome editing of plants in the T0 generation is especially useful for vegetatively propagated and perennial plant species. Compared to transgenic plants, transgene-free genome edited plants have multiple promising properties: 1) Easier path for deregulation and commercialization. Both USDA Animal & Plant Health Inspection Service (APHIS) and US Environmental Protection Agency exempt transgene-free genome-edited plants (Turnbull et al., 2021; Su et al., 2023), 2) Alleviate the potentially deleterious effects from the T-DNA integrated into the host genome stably (O’Malley and Ecker, 2010), 3) Reducing off-target mutations by eliminating the constitutive expression of genome editing systems. Off-target mutations are another critical factor for consideration during genetic improvement by genome editing. Transient expression of Cas/gRNA DNA, mRNA, and RNP in embryogenic protoplasts, calli, or immature embryo cells has been reported to generate transgene-free plants without causing off-target mutations (Woo et al., 2015; Zhang et al., 2016; Liang et al., 2017). This probably results from the short functional time of Cas/gRNA during transient expression, as suggested by previous studies (Huang et al., 2022a; Randall et al., 2021). In addition to the co-editing method, transformation of citrus embryogenic protoplast cells has also been successfully used to generate transgene-free canker-resist Hamlin by editing *CsLOB1* coding region (Su et al., 2023). It is noteworthy that our work here targeted *CsLOB1* promoter elements, EBE_PthA4_-LOBP. Even though editing either the coding region or promoter region of *LOB1* generates canker-resistant citrus varieties, it remains to be determined whether there are any phenotypical changes between the two different editions.

In summary, our improved co-editing approach provides a cost-effective, time-saving and one-step method to produce transgene-free genome-edited citrus in the T0 generation. This strategy has the potential to be expanded to other plant species, especially those that have long juvenility or/and must be produced through vegetative propagation.

## Data availability statement

The datasets presented in this study can be found in online repositories. The names of the repository/repositories and accession number(s) can be found below: https://www.ncbi.nlm.nih.gov/genbank/, PRJNA1073671.

## Author contributions

HJ: Investigation, Methodology, Writing – original draft, Writing – review & editing. AO: Methodology, Resources, Writing – review & editing. JX: Data curation, Investigation, Methodology, Writing – original draft, Writing – review & editing. JD: Investigation, Writing – review & editing. YW: Investigation, Writing – review & editing. YF: Investigation, Writing – review & editing. WW: Investigation, Writing – review & editing. ZH: Investigation, Writing – review & editing. JG: Resources, Writing – review & editing. NW: Conceptualization, Funding acquisition, Investigation, Project administration, Supervision, Writing – original draft, Writing – review & editing.

## Supplementary material

Supplementary Figure 6 Whole genome sequencing analysis of #Ham_NoGFP_4. **(A)** Based on whole genome sequencing, two alleles of *CsALS* of #Ham_NoGFP_4 contained the identical 6^th^, 7^th^, 8^th^ C->T mutations. **(B)** As for EBE_PthA4_-LOBP of #Ham_NoGFP_4, there was 7 bp deletion of CCTTTTG from EBE region of Type I and Type II allele. The mutations were showed by horizontal bar chart. The vertical bar chart showed the sequence depth for each base.
