# Supplementary material for: Generation of transgene-free canker-resistant Citrus sinensis cv. Hamlin in the T0 generation through Cas12a/CBE co-editing
Source: Front Plant Sci. 2024 Mar 26;15:1385768. doi: 10.3389/fpls.2024.1385768 (PMC11002166; doi:10.3389/fpls.2024.1385768)
Supplement: Supplementary Figure 6 — Whole genome sequencing analysis of #HamNoGFP4. (A) Based on whole genome sequencing, two alleles of CsALS of #HamNoGFP4 contained the identical 6th, 7th, 8th C->T mutations. (B) As for EBEPthA4-LOBP of #HamNoGFP4, there was 7 bp deletion of CCTTTTG from EBE region of Type I and Type II allele. The mutations were showed by horizontal bar chart. The vertical bar chart showed the sequence depth for each base. [file DataSheet_1.pdf]

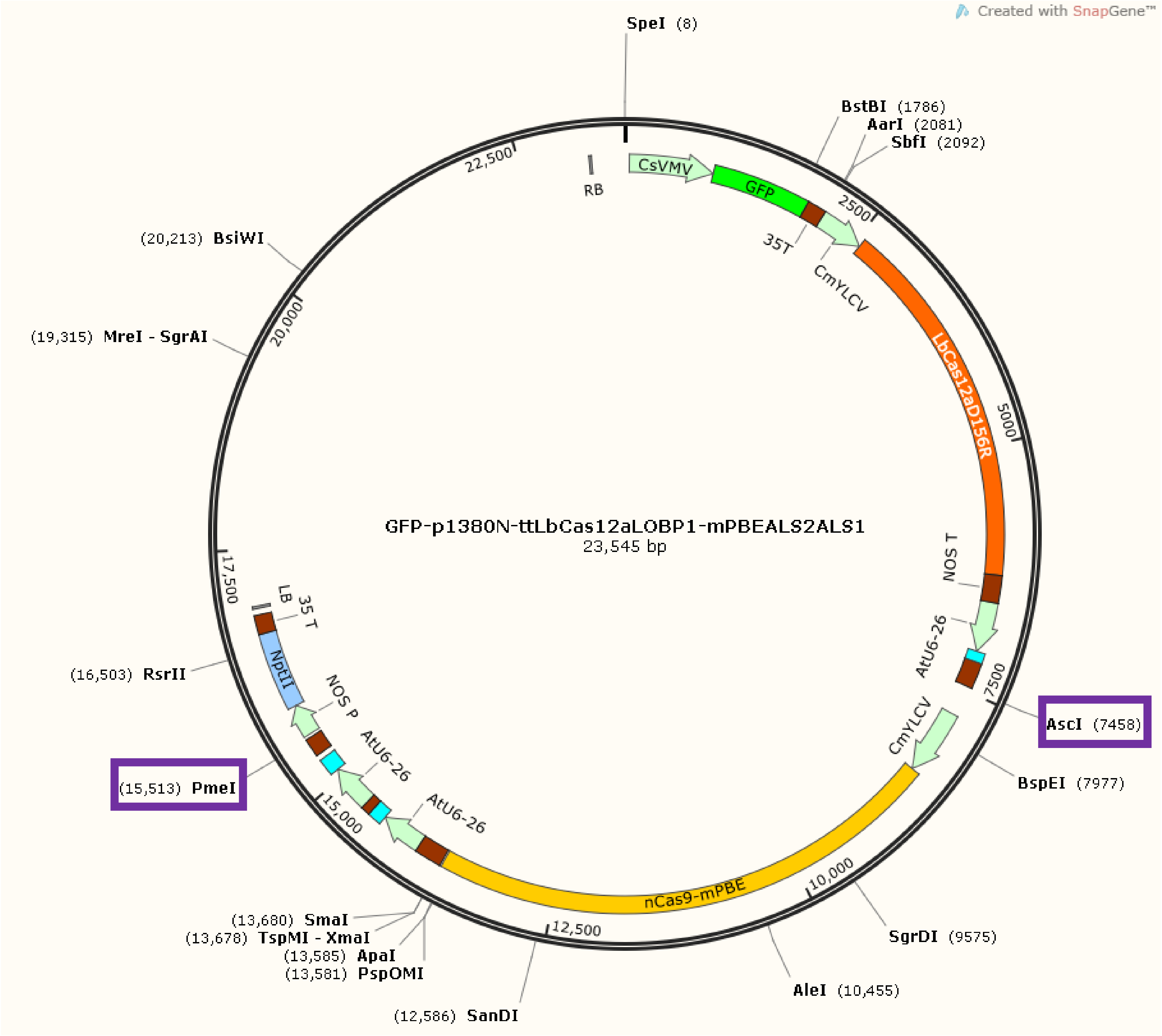

Supplementary Figure 1. Schematic map of the binary vector GFP-p1380N-ttLbCas12a:LOBP1-mPBE:ALS2:ALS1. Enzymes *Asc*I and *Pme*I were employed to clone mPBE:ALS2:ALS1 fragment into *Asc*I-*Pme*I-cut GFP-p1380N-ttLbCas12a:LOBP1-*Asc*I-*Xba*I-*Pme*I to build GFP-p1380N-ttLbCas12a:LOBP1-mPBE:ALS2:ALS1. *Asc*I and *Pme*I were highlighted by purple rectangles.

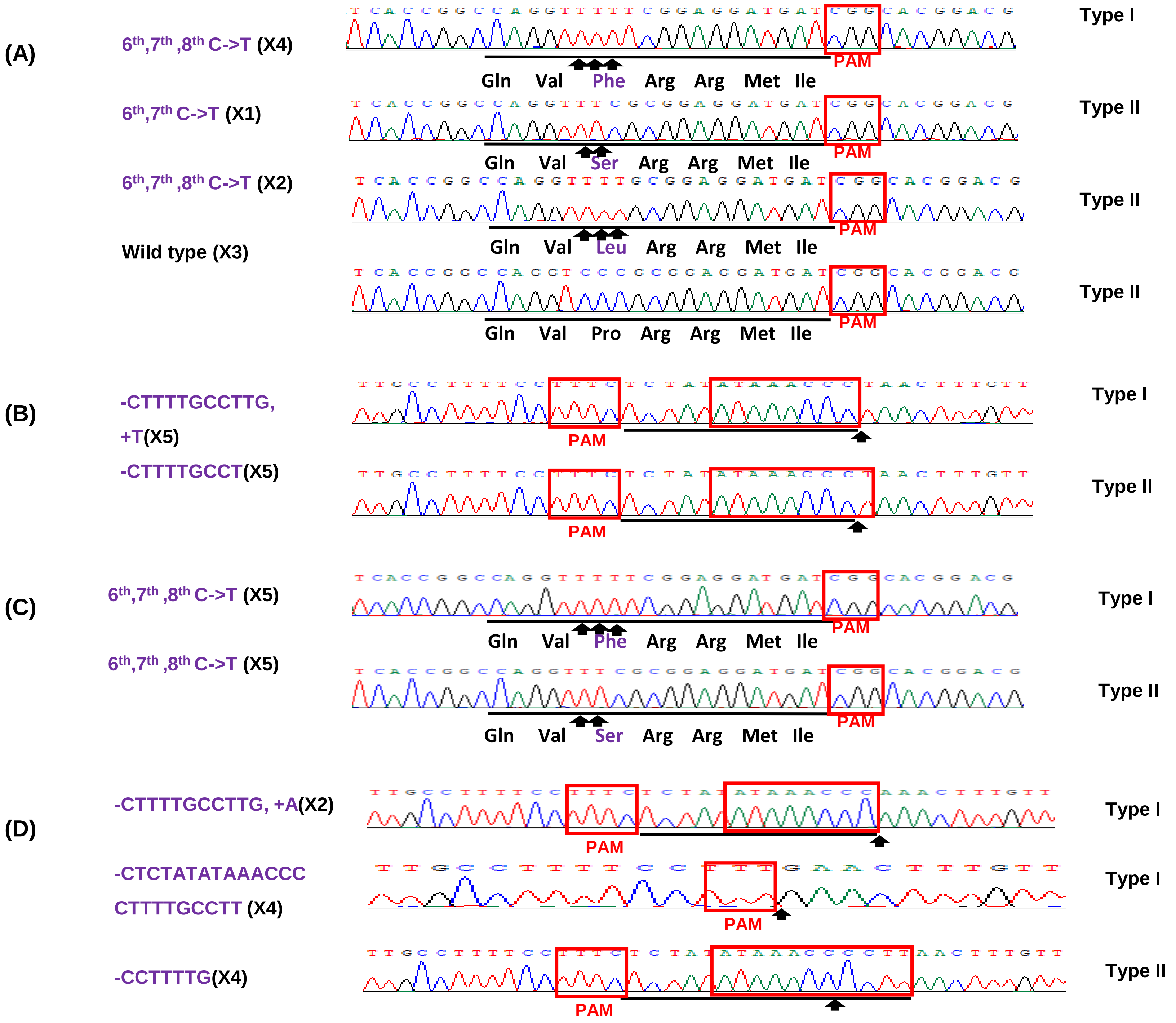

Supplementary Figure 2. Sanger sequencing results of #Ham$_{GFP}$1 and #Ham$_{GFP}$2. (A) As for *CsALS* of #Ham$_{GFP}$1, there were 6th, 7th C->T conversions, 6th, 7th, 8th C->T changes and wild type among 10 colonies sequenced. (B) As for EBE$_{PthA4}$-LOBP of #Ham$_{GFP}$1, Type I allele had CTTTTGCCTTG deletion / T insertion, and Type II allele contained CTTTTGCCT deletion. (C) As for *CsALS* of #Ham$_{GFP}$2, all of them were base editing occurred at 6th, 7th, 8th C->T among 10 colonies sequenced. (D) As for EBE$_{PthA4}$-LOBP of #Ham$_{GFP}$2, Type I allele had CTTTTGCCTTG deletion / A insertion and CTCTATATAAACCCCTTTTGCCTT, and Type II allele harbored CCTTTTG deletion.

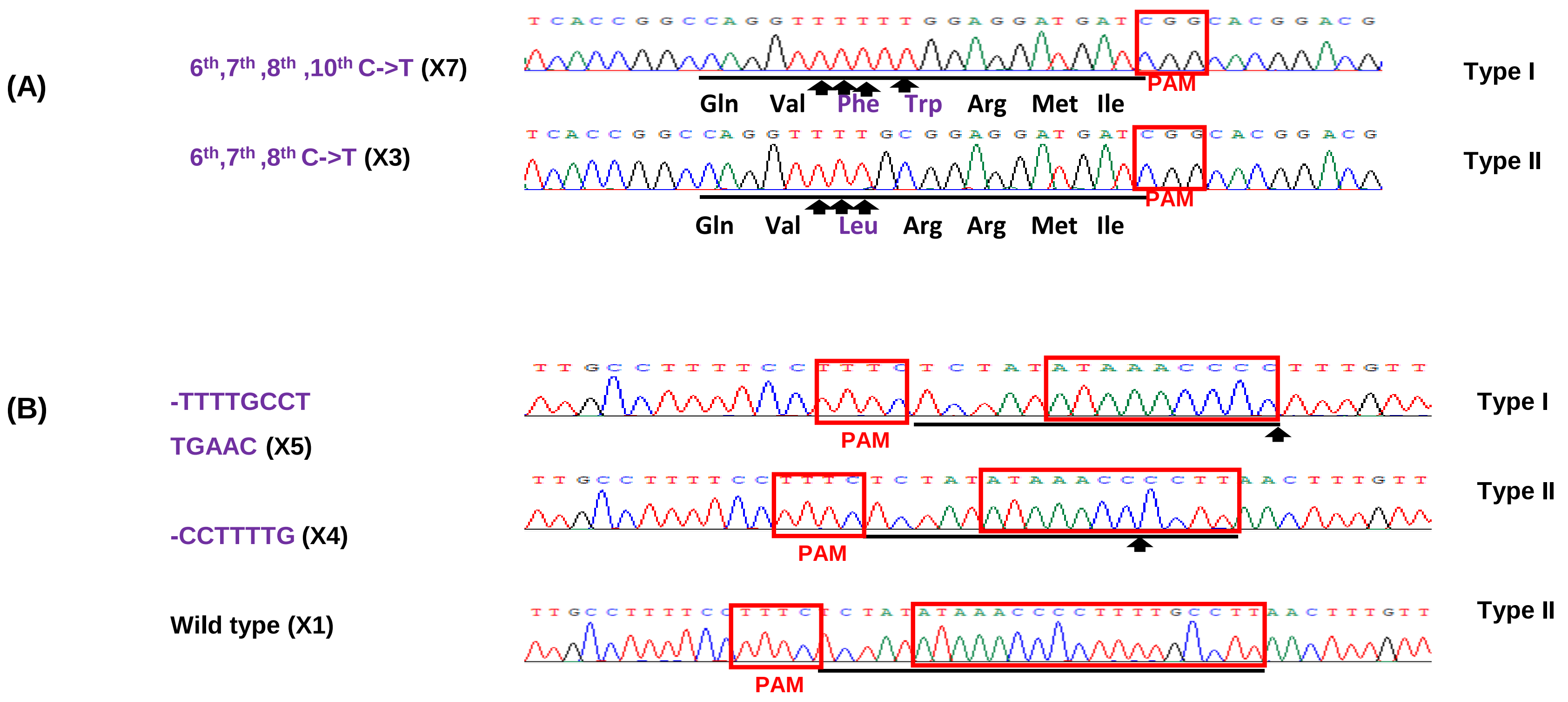

Supplementary Figure 3. Sanger sequencing results of #Ham$_{GFP}$3. (A) As for *CsALS* of #Ham$_{GFP}$3, 6th,7th ,8th,10th C->T and 6th,7th ,8th C->T changes were present among 10 colonies sequenced. (B) As for EBE$_{PthA4}$-LOBP of #Ham$_{GFP}$3, Type I allele contained TTTTGCCTTGAAC deletion, and Type II allele had CCTTTTG deletion and wild type.

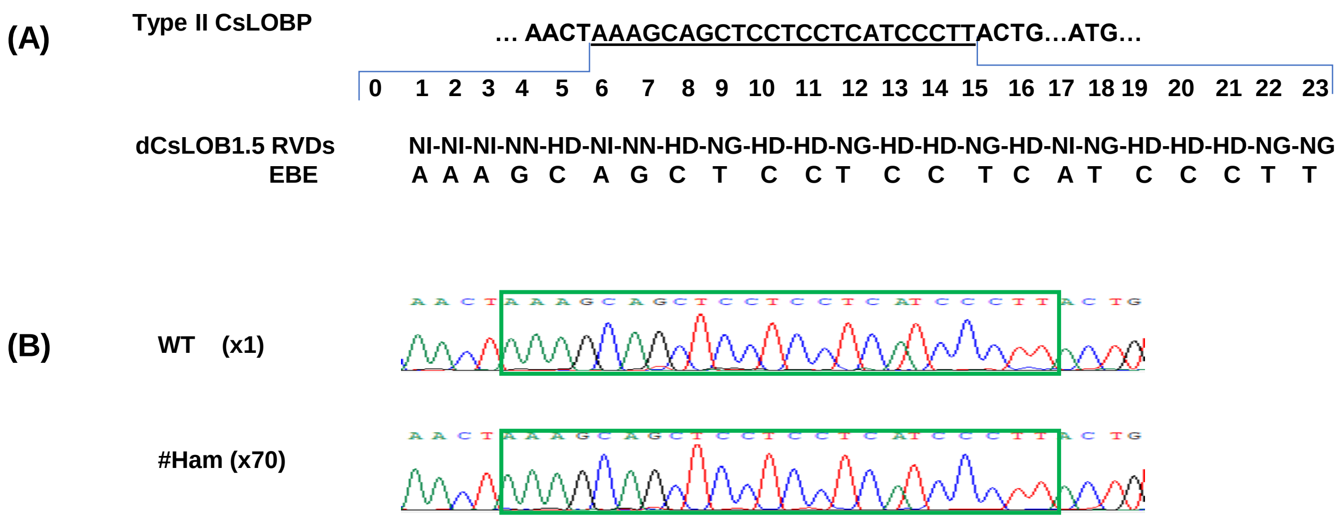

Supplementary Figure 4. dCsLOB1.5 and its representative chromatograms in Hamlin. (A) dCsLOB1.5 is an artificial dTALE, which specifically recognizes AAAGCAGCTCCTCCTCATCCCTT downstream of EBE_{PthA4}-TI LOBP by 16 bp and EBE_{PthA4}-TII LOBP by 15 bp. (B) Representative chromatograms of dCsLOB1.5-binding sequence, which has no indels in wild type, transgenic and transgene-free Hamlin plants. The dCsLOB1.5-binding sequence is highlighted by green rectangles.

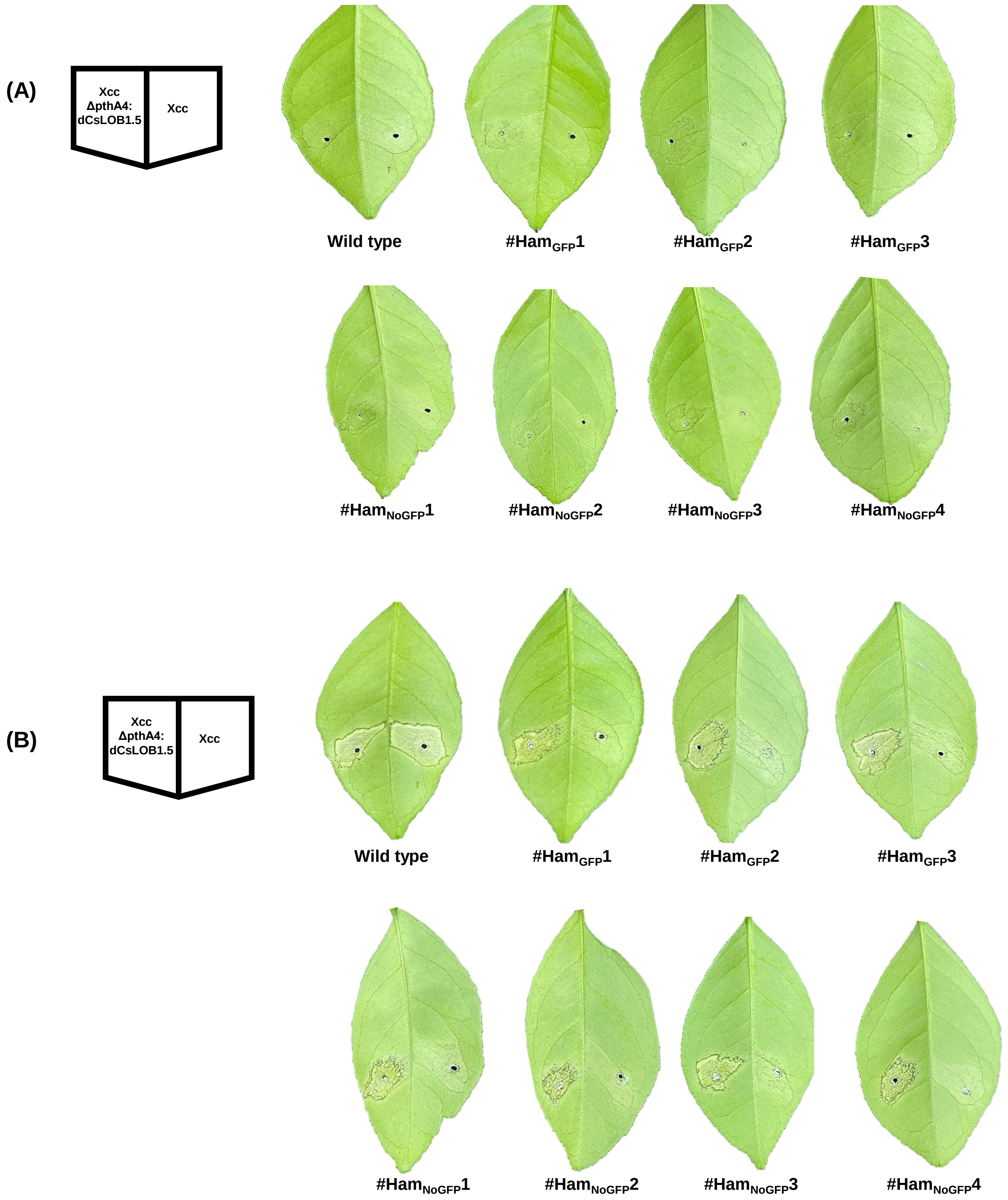

Supplementary Figure 5. Canker-resistance in the transgenic and transgene-free EBE$_{PthA4}$-LOBP-edited Hamlin plants at three days post Xcc inoculation (DPI) (A) and at 9 DPI (B). (A) At 3 DPI, citrus canker symptoms started to appear on wild type Hamlin, whereas no canker symptoms were observed on LOBP-edited Hamlin plants. As expected, *XccpthA4:Tn5* (dCsLOB1.5)-inducing canker symptoms started to appear on all plants. (B) At 9 DPI, citrus canker symptoms were observed on wild type Hamlin, whereas no canker symptoms were observed on LOBP-edited Hamlin plants. As expected, *XccpthA4:Tn5* (dCsLOB1.5) caused canker symptoms on all plants.

(A)

(B)

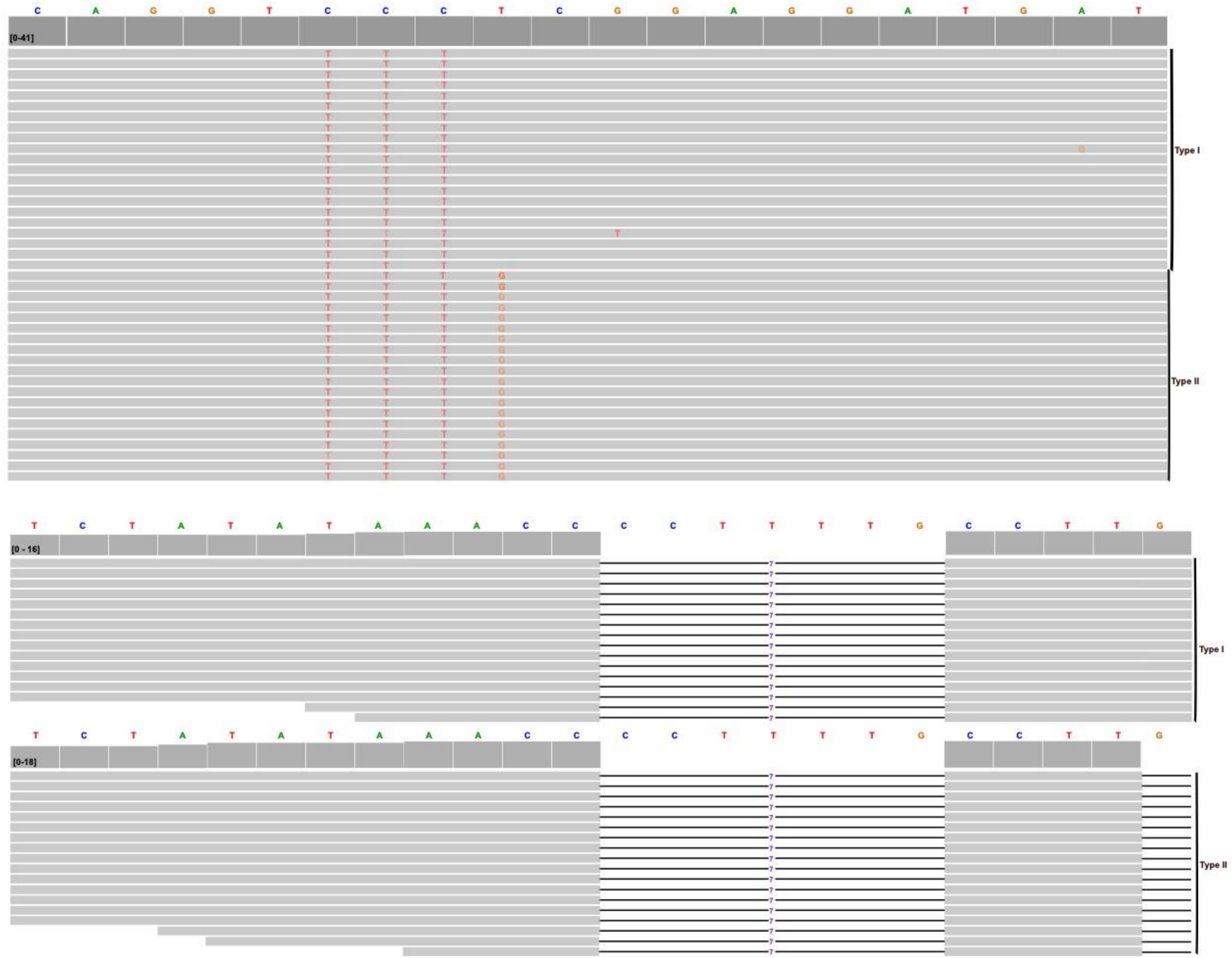

Supplementary Figure 6. Whole genome sequencing analysis of #Ham$_{NoGFP}$4. (A) Based on whole genome sequencing, two alleles of *CsALS* of #Ham$_{NoGFP}$4 contained the identical 6th, 7th, 8th C->T mutations. (B) As for EBE$_{PthA4}$-LOBP of #Ham$_{NoGFP}$4, there was 7 bp deletion of CCTTTTG from EBE region of Type I and Type II allele. The mutations were showed by horizontal bar chart. The vertical bar chart showed the sequence depth for each base.
